# BivRec: an R package for the nonparametric and semiparametric analysis of bivariate alternating recurrent events

**DOI:** 10.1186/s12874-022-01558-0

**Published:** 2022-04-03

**Authors:** Sandra Castro-Pearson, Aparajita Sur, Chi Hyun Lee, Chiung-Yu Huang, Xianghua Luo

**Affiliations:** 1 Division of Biostatistics, School of Public Health, University of Minnesota, Minneapolis, USA; 2 Department of Biostatistics and Epidemiology, School of Public Health and Health Science, University of Massachusetts Amherst, Amherst, USA; 3 Department of Epidemiology and Biostatistics, University of California San Francisco, San Francisco, USA; 4 Biostatistics Core, Masonic Cancer Center, University of Minnesota, Minneapolis, USA

**Keywords:** BivRec, Bivariate gap times, Recurrent events

## Abstract

**Background:**

Bivariate alternating recurrent event data can arise in longitudinal studies where patients with chronic diseases go through two states that occur repeatedly, e.g., care periods and break periods. However, there was no statistical software that provided tools for the analysis of such data. To meet this software need, we developed BivRec, a package for R that contains a set of tools for exploratory, nonparametric and semiparametric regression analysis of bivariate alternating recurrent events.

**Results:**

The BivRec package provides functions for nonparametric estimations for the joint distribution of bivariate gap times (bivrecNP) and semiparametric regression methods for evaluating covariate effects on the two types of gap times under the accelerated failure time model framework (bivrecReg). The package also provides exploratory data analysis tools such as a visualization of the gap times by groups. We utilize a subset of the South Verona Psychiatric Case Register (PCR) data to illustrate the use of the BivRec package for the reviewed methods.

**Conclusions:**

We demonstrate BivRec’s capability for data visualization, nonparametric and regression based analysis, as well as data simulation. The package has default methods with satisfactory performance despite the complexity of calculations and fills a gap in software for statistical analysis of bivariate alternating recurrent events. BivRec is accessible under the GPL-3 General Public License through CRAN, facilitating its installation.

## Background

Bivariate alternating recurrent event data can arise in longitudinal studies where individuals go through two interchanging states that reoccur over time. For instance, patients with chronic conditions, such as addiction or mental illnesses, experience care periods and break periods repeatedly. The on and off periods of care or disease form a sequence of recurrent gap times. Consider a group of patients who enter a study at the time of a hospitalization due to a particular disease and during the study experience a series of recurrent hospitalizations caused by the same disease. In this case, patients experience alternating periods of care and breaks from care (referred to as *Type I* and *Type II* gap times, respectively, hereinafter) until a censoring event such as the end of study occurs.

First, we introduce some notation. Let $X^{0}_{ij}$ and $Y^{0}_{ij}$ be random variables representing the length of Type I and Type II gap times of episode *j* experienced by subject *i*, respectively. Denote the collection of all episodes of subject *i* by $N_{i}= \left \{\left (X^{0}_{i1}, Y^{0}_{i1}\right), \left (X^{0}_{i2}, Y^{0}_{i2}\right), \ldots \right \}, i=1, 2, \ldots, n$. In addition, we assume that the bivariate recurrent event process of subject *i* is subject to right censoring *C*_*i*_, which has survival function *G*(·) with maximum support *τ*_*c*_= sup{*t*:*G*(*t*)>0}. Let *m*_*i*_ denote the number of episodes of bivariate alternating recurrence times for subject *i*, which satisfies the conditions:

$$\sum\limits_{j=1}^{m_{i}-1} (X^{0}_{ij} + Y^{0}_{ij}) \le C_{i} \text{and} \sum\limits_{j=1}^{m_{i}} (X^{0}_{ij} + Y^{0}_{ij}) > C_{i}. $$

Note that, in the last bivariate pair $\left (X^{0}_{{im}_{i}},Y^{0}_{{im}_{i}}\right)$, $X^{0}_{{im}_{i}}$ may or may not be censored, but $Y^{0}_{{im}_{i}}$ is always censored, for which we use the hospitalization example to illustrate. When the censoring occurs during the last care period, $X_{{im}_{i}}^{0}$, the break period afterwards, $Y^{0}_{{im}_{i}}$ cannot be observed, and when the censoring occurs during the last break period, $Y^{0}_{{im}_{i}}$ is only partially observed.

**Fig. 1:**
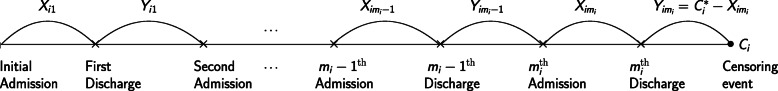
Illustration of a bivariate alternating recurrent event process

The observation of the gap times $X^{0}_{ij}$ and $Y^{0}_{ij}$ is subject to the censoring time $C_{ij}^{*} = C_{i} - \sum \limits _{l=1}^{j-1} \left (X^{0}_{il} + Y^{0}_{il}\right)$ and $\max \left \{C_{ij}^{*}-X_{ij}^{0},0\right \}$, respectively, where $\sum \limits _{1}^{0}=0$. Due to the censoring, the observed data for subject *i* are $\left \{\left (X_{ij}, Y_{ij}, \Delta ^{X}_{ij}, \Delta ^{Y}_{ij}\right),j=1,\ldots,m_{i}\right \}$, where $X_{ij}= X_{ij}^{0}, Y_{ij} = Y_{ij}^{0}$ and $\Delta ^{X}_{ij}=\Delta ^{Y}_{ij}=1$ for *j*=1,…,*m*_*i*_−1, while $X_{{im}_{i}} = {\min }\left (X_{{im}_{i}}^{0}, C_{{im}_{i}}^{*}\right), Y_{{im}_{i}} = \min \left \{Y_{{im}_{i}}^{0}, \max \left (C_{{im}_{i}}^{*}-X_{{im}_{i}}, 0\right)\right \}, \Delta ^{X}_{{im}_{i}}= I\left (X_{{im}_{i}}^{0} < C_{{im}_{i}}^{*}\right)$ and $\Delta ^{Y}_{{im}_{i}}=0$. Figure 1 shows an illustration of this process where the Type I gap of the last episode is observed but the Type II gap following it is censored (i.e., $0 < C_{{im}_{i}}^{*}-X_{{im}_{i}} < Y_{{im}_{i}}^{0}$).

The BivRec package was designed to analyze bivariate alternating recurrent data with the form as depicted in Fig. 1. It provides a consistent and user-friendly set of functions to: explore and visualize the data, estimate and plot the joint cumulative distribution function (*cdf*), the marginal survival and the conditional *cdf* using nonparametric methods [1] and fit semiparametric accelerated failure time models [2, 3] to estimate the effect of covariates on the two alternating gap times.

BivRec version 1.2.1 is available through CRAN at https://cran.r-project.org/package=BivRec. The reference manual can be found in both CRAN and Additional file 1 of this paper. The implementation in R was designed for users with some experience analyzing survival data and follows conventions used in similar R packages. Most functions in BivRec are S4 methods that produce S4 class objects. Where possible functions have been optimized using Fortran 90 to reduce running time.

## Implementation

### Nonparametric analysis

In 2005, Huang and Wang [1] developed a nonparametric method for estimating the joint distribution of the two types of alternating gap times, which is a useful data summary tool for bivariate alternating recurrent event data. In the BivRec package, we implemented Huang and Wang’s (2005) [1] nonparametric methods in the function bivrecNP() to estimate:

1. the joint cumulative distribution function (*cdf*) for the two types of gap times, $\Pr \left (X_{ij}^{0} \leq x, Y_{ij}^{0} \leq y\right)$, and its associated standard error (in an output data frame and a contour plot),
2. the marginal survival function for the Type I gap times, $\Pr \left (X_{ij}^{0} > x\right)$, and its associated standard error (in an output data frame and a survival plot), and
3. the conditional *cdf* for the Type II gap times given the Type I gap times, $\Pr \left (Y_{ij}^{0} \leq y|X_{ij}^{0} \leq x\right)$, and its associated standard error (in an output data frame and a conditional *cdf* plot).

For estimation for the *cdf*, assume that there exists a subject-level latent variable *Ψ*_*i*_ with an unspecified *cdf*, *P*_*Ψ*_(·) such that the bivariate gap times $\left (X^{0}_{ij}, Y^{0}_{ij}\right), j=1, 2,\ldots $ are identically and independently distributed (*i.i.d.*) given *Ψ*_*i*_ and that the censoring time *C*_*i*_ is independent of (*N*_*i*_,*Ψ*_*i*_). Define variables $Z^{0}_{ij}= X^{0}_{ij} + Y^{0}_{ij}$ and $\boldsymbol {W}^{0}_{ij} = \left (X^{0}_{ij}, Y^{0}_{ij}\right)$, then their joint *cdf* is $F_{Z^{0}, \boldsymbol {W}^{0}}( {z}, \boldsymbol {u})=\Pr \left (X^{0}_{i1} + Y^{0}_{i1} \leq  {z}, X^{0}_{i1} \leq x, Y^{0}_{i1} \leq y\right)$ for *z*=*x*+*y* and ***u***=(*x*,*y*). The marginal survival function of $Z^{0}_{ij}$ is hence $S_{Z^{0}}( {z}) = 1 - F_{Z^{0}, \boldsymbol {W}^{0}}( {z}, (\infty, \infty))$. Our interest lies in the estimation of the joint distribution $F_{X^{0}, Y^{0}}(x, y)= \Pr (X^{0}_{ij} \leq x, Y^{0}_{ij} \leq y)$, which is determined by $F_{Z^{0}, \boldsymbol {W}^{0}}( {z}, \boldsymbol {u})$ through the identity $F_{X^{0}, Y^{0}}(x, y) = F_{Z^{0}, \boldsymbol {W}^{0}}(x+y, (x,y))$. Let *F*_*a*_(*z*,***u***)=E[*a*_*i*_*I*(*Z*_*i*1_≤*z*,***W***_*i*1_≤***u***,*Δ*_*i*1_=1)] and *R*_*a*_(*z*)=E[*a*_*i*_*I*(*Z*_*i*1_≥*z*)], where the weight *a*_*i*_=*a*(*C*_*i*_) is a non-negative function of *C*_*i*_ and satisfies $\mathrm {E}[a_{i}^{2}]<\infty $, with a special case of *a*_*i*_=1, i.e., no weights. Then, following Huang and Louis (1998) [4], it is shown that $F_{Z^{0}, \boldsymbol {W}^{0}}( {z}, \boldsymbol {u}) = \int _{0}^{ {z}} S_{Z^{0}}(s-) \frac {F_{a}(ds,\boldsymbol {u})}{R_{a} (s)}$, where $S_{Z^{0}}(\cdot)$ is the marginal survival function of $Z^{0}_{ij}$ [1]. Noticing that *F*_*a*_(*ds*,***u***) and *R*_*a*_(*s*) can be replaced with their respective empirical estimators and that the survival function $S_{Z^{0}}(\cdot)$ can be estimated by the estimator of [5] for univariate (i.e., single-type) recurrent gap times $Z^{0}_{ij}$, one can estimate the joint distribution of interest for any (*x*,*y*) satisfying *x*+*y*≤*τ*_*c*_ as follows:

1 $$ {{\begin{aligned} \hat{F}_{X^{0}, Y^{0}}(x,y) = \sum\limits_{t_{k}^{*} \leq x+y} \prod_{l< k} \left\{1- \frac{\hat{H}_{a}(t_{l}^{*}, \infty)}{\hat{R}_{a}(t_{l}^{*})}\right\} \frac{\hat{H}_{a}(t_{k}^{*}, (x,y))}{\hat{R}_{a}(t_{k}^{*})},  \end{aligned}}}  $$

where $t_{1}^{*}, t_{2}^{*}, \dots t_{K}^{*}$ are the distinct and uncensored recurrence times from $\{Z_{ij}, j= 1, \dots m_{i}^{*}, i=1, \dots, n\}$ with $m_{i}^{*} = m_{i} - 1$ for *m*_*i*_≥2 and $m_{i}^{*}=1$ for *m*_*i*_=1, $\hat {H}_{a}( {z},\boldsymbol {u}) = n^{-1} \sum \limits _{i=1}^{n} \frac {a_{i} I(m_{i} \geq 2)}{m_{i}^{*}} \sum \limits _{j=1}^{m_{i}^{*}}I(Z_{ij} =  {z}, \boldsymbol {W}_{ij} \leq \boldsymbol {u})$ and $\hat {R}_{a}( {z}) = n^{-1} \sum \limits _{i=1}^{n} \frac {a_{i}}{m_{i}^{*}} \sum \limits _{j=1}^{m_{i}^{*}} I(Z_{ij} \geq  {z})$.

Next, we focus on the estimation of the marginal survival function for Type I gap times, $S_{X^{0}}(x)=\Pr (X^{0}_{i1}\ge x)$. Let $x_{1}^{*}, x_{2}^{*}, \dots x_{M}^{*}$ denote the distinct and uncensored Type I gap times from $\{X_{ij}, j= 1, \dots m_{i}^{*}, i=1, \dots, n\}$. Following Wang and Chang (1999) [5], Huang and Wang in 2005 [1] proposed to estimate $S_{X^{0}}(x)$ for *x*≤*τ*_*c*_ with:

$$\hat{S}_{X^{0}}(x) = \prod_{x^{*}_{k} \leq x} \left\{1- \frac{\hat{H}_{X}(x^{*}_{k})}{\hat{R}_{X}(x^{*}_{k})}\right\}, $$

where $\hat {H}_{X}(t) = \frac {1}{n} \sum \limits _{i=1}^{n} \frac {a_{i} I(m_{i} \geq 2)}{m_{i}^{*}} \sum \limits _{j=1}^{m_{i}^{*}}I(X_{ij} = t)$ and $\hat {R}_{X}(t) = \frac {1}{n} \sum \limits _{i=1}^{n} \frac {a_{i}}{m_{i}^{*}} \sum \limits _{j=1}^{m_{i}^{*}} I(X_{ij} \geq t).$ Finally, they argued that the marginal distribution of Type II gap times $Y^{0}_{ij}$ is not estimable due to induced dependent censoring, and hence proposed to estimate the conditional distribution of Type II gap times given Type I gap times, $F_{Y^0|X^{0}}(y|x) = \Pr (Y^{0}_{i1} \leq y | X^{0}_{i1} \leq x)$ using the estimator

$$\hat{F}_{Y^0|X^{0}}(x,y) = \frac{\hat{F}_{X^{0}, Y^{0}}(x,y)}{1 - \hat{S}_{X^{0}}(x)}, \text{for}\ x+y \leq \tau_{c}. $$

Similarly, one can estimate $\Pr (Y^{0}_{i1} \leq y |x_{2}\leq X^{0}_{i1} \leq x_{1})$ by $ \frac {\hat {F}_{X^{0}, Y^{0}}(x,y)}{\hat {S}_{X^{0}}(x_{1}) - \hat {S}_{X^{0}}(x_{2})}$ for *x*_1_+*y*≤*τ*_*c*_.

The standard errors of the estimators for the joint distribution and the marginal survival function of Type I gap times are estimated based on the large sample properties of these estimators proved in [1]. Briefly, for 0≤*x*+*y*≤*L*,*L*<*τ*<*τ*_*c*_ with *τ* being the maximal support of *R*_*a*_(*z*), $\sqrt {n}\{\hat {F}_{X^{0}Y^{0}}(x,y) - F_{X^{0}Y^{0}}(x,y)\}$ weakly converges to a Gaussian process with mean zero and variance-covariance function *Σ*=E[*ϕ*_1_(*z*_1_,***u***_1_)*ϕ*_1_(*z*_2_,***u***_2_)]. Similarly, $\sqrt {n} \{\hat {S}_{x^{0}}(x) - S_{x^{0}}(x)\}$ converges weakly to a Gaussian process with mean zero and with variance-covariance function $\Sigma _{s} = S_{x^{0}}(x_{1})S_{x^{0}}(x_{2})\mathrm {E}[\xi _{1}(x_{1})\xi _{1}(x_{2})]$ where *x*_1_,*x*_2_∈[0,*L*]. The definitions of *ϕ*_1_(*z*,***u***) and *ξ*_1_(*x*) can be found in [1]. For the conditional distribution estimator, $\hat F_{Y^{0}|X^{0}}(y|x)$, the package provides the bootstrap standard error and confidence intervals.

### Semiparametric regression

Researchers, especially in clinical settings, are often more interested in regression methods that allow them to understand the relationship between covariates and the recurrent event process. In this regard, Chang in 2004 [2] proposed a semiparametric accelerated failure time (AFT) model which allows the estimation of the covariate effects on the two types of alternating gap times to be done simultaneously (referred to as *Chang’s method* hereinafter). Recognizing that the estimation of the AFT model coefficients by Chang is based on a nonsmooth, rank-based estimating function, Lee et al. in 2018 [3] proposed a smooth, U-statistic-based estimating function whose solution is found to be more computationally tractable (referred to as *Lee et al.’s method* hereinafter). We now briefly review the AFT model for bivariate recurrent gap times and the estimation methods developed by [2] and [3] and implemented in the function bivrecReg().

Let ***A***_*i*_ denote a *p*×1 vector of baseline covariates of subject *i* and ***γ***_*i*_=(*γ*_*i*1_,*γ*_*i*2_)^′^, a subject-specific latent vector that carries information for within subject correlations among the recurrent gap times. The censoring time *C*_*i*_ is assumed to be independent of (*N*_*i*_,***A***_*i*_,***γ***_*i*_). The AFT model assumes that conditioning on ***A***_*i*_ and ***γ***_*i*_, the bivariate gap time pairs $(X_{ij}^{0},Y_{ij}^{0}),j=1,2,\ldots $ are *i.i.d.* within subject *i*. Furthermore, each (log) gap time is linearly related to the covariates as follows:

2 $$\begin{array}{@{}rcl@{}} \log X_{ij}^{0} &=& \gamma_{i1} + \boldsymbol{A}_{i}'\boldsymbol{\beta}_{1} + \varepsilon_{ij1},\\ \log Y_{ij}^{0} &=& \gamma_{i2} + \boldsymbol{A}_{i}'\boldsymbol{\beta}_{2} + \varepsilon_{ij2}, \end{array} $$

where ***β***_1_,***β***_2_ denote the regression coefficients for Type I and Type II gap times, respectively, and *ε*_*ijk*_,*k*=1,2, are mutually independent random errors with mean zero. Both the errors and the latent vectors ***γ***_*i*_ come from unspecified distributions.

In 2004, Chang [2] considered the transformed, complete gap times given by $\tilde {X^{0}_{ij}}(\boldsymbol {b})=X^{0}_{ij}\exp (-\boldsymbol {A}_{i}^{\prime } \boldsymbol {b}_{1})$ and $\tilde {Z^{0}_{ij}}(\boldsymbol {b})=X^{0}_{ij}\exp (-\boldsymbol {A}_{i}^{\prime } \boldsymbol {b}_{1})+Y^{0}_{ij}\exp (-\boldsymbol {A}_{i}^{\prime } \boldsymbol {b}_{2})$, where $\boldsymbol {b}=(\boldsymbol {b}_{1}^{\prime },\boldsymbol {b}_{2}^{\prime })^{\prime }$. Their observed counterparts are:

$${{\begin{aligned} \tilde{X}_{ij}(\boldsymbol{b})=\min\left\{\tilde{X}^{0}_{ij}(\boldsymbol{b}),C_{i} \exp(-\boldsymbol{A}^{\prime}\boldsymbol{b}_{1})-\sum\limits_{l=1}^{j-1}\tilde{Z}_{il}(\boldsymbol{b})\right\}, \text{and} \end{aligned}}} $$

$$\tilde{Z}_{ij}(\boldsymbol{b})=\min\left\{\tilde{Z}^{0}_{ij}(\boldsymbol{b}),C_{i} \exp(-\boldsymbol{A}^{\prime}\boldsymbol{b}_{1})-\sum\limits_{l=1}^{j-1}\tilde{Z}_{il}(\boldsymbol{b})\right\}. $$

The rank-based estimating functions [2] are:

$$\begin{array}{@{}rcl@{}} U_{1}(\boldsymbol{b}) &=& n^{-1/2} \sum\limits_{i=1}^{n}\frac{1}{m_{i}^{*}}\sum\limits_{j=1}^{m_{i}^{*}} \left[\boldsymbol{A}_{i}-\frac{S_{1}^{X}\{\boldsymbol{b},\tilde{X}_{ij}(\boldsymbol{b})\}}{S_{0}^{X}\{\boldsymbol{b},\tilde{X}_{ij}(\boldsymbol{b})\}}\right],\\ U_{2}(\boldsymbol{b}) &=& n^{-1/2} \sum\limits_{i=1}^{n}\frac{1}{m_{i}^{*}}\sum\limits_{j=1}^{m_{i}^{*}} \left[\boldsymbol{A}_{i}-\frac{S_{1}^{Z}\{\boldsymbol{b},\tilde{Z}_{ij}(\boldsymbol{b})\}}{S_{0}^{Z}\{\boldsymbol{b},\tilde{Z}_{ij}(\boldsymbol{b})\}}\right], \end{array} $$

where $S_{k}^{X}(\boldsymbol {b},t) = n^{-1}\sum \limits _{i=1}^{n}\frac {1}{m_{i}^{*}}\sum \limits _{j=1}^{m_{i}^{*}}\boldsymbol {A}_{i}^{\otimes k}I(\tilde {X}_{ij}(\boldsymbol {b})\ge t)$ and $S_{k}^{Z}(\boldsymbol {b},t) = n^{-1}\sum \limits _{i=1}^{n}\frac {1} {m_{i}^{*}}\sum \limits _{j=1}^{m_{i}^{*}}\boldsymbol {A}_{i}^{\otimes k}\cdot I(\tilde {Z}_{ij}(\boldsymbol {b})\ge t)$ for *k*=0,1 and $\boldsymbol {A}_{i}^{\otimes k} =1$ for *k*=0; $\boldsymbol {A}_{i}^{\otimes k} = \boldsymbol {A}_{i}$ for *k*=1. Denote the solution to *U*_1_(***b***)=0 and *U*_2_(***b***)=0 by $\hat {\beta }_{\text {Chang}}$. Chang (2004) proposed to use the resampling method in [6] to estimate the covariance matrix for $\hat {\boldsymbol {\beta }}_{\text {Chang}}$. Since both the point estimate and the resampling-based interval estimate rely on solving nonsmooth estimating functions, fitting the AFT model with Chang’s method can be computationally inefficient and even encounter a nonconvergence problem, in which case the R function will give the user an error message.

Motivated by a multi-state model [7], Lee et al. [3] defined the transformed gap times $X^{0}_{ii^{\prime }j}(\boldsymbol {b}_{1}) = \exp \left (\boldsymbol {A}_{ii^{\prime }}^{\prime }\boldsymbol {b}_{1}\right)X_{ij}^{0}$ and $Z^{0}_{ii^{\prime }j}(\boldsymbol {b}) = \exp \left (\boldsymbol {A}_{ii^{\prime }}^{\prime }\boldsymbol {b}_{1}\right)X_{ij}^{0} + \exp \left (\boldsymbol {A}_{ii^{\prime }}^{\prime }\boldsymbol {b}_{2}\right)Y_{ij}^{0}$ with $\boldsymbol {A}_{ii^{\prime }} = \boldsymbol {A}_{i^{\prime }}-\boldsymbol {A}_{i}$. Their observed counterparts are:

$$X_{ii^{\prime}j}(\boldsymbol{b}_{1}) = \exp(\boldsymbol{A}_{ii^{\prime}}^{\prime}\boldsymbol{b}_{1})X_{ij},\ \text{and} $$

$$Z_{ii^{\prime}j}(\boldsymbol{b}) \,=\, \exp(\boldsymbol{A}_{ii^{\prime}}^{\prime}\boldsymbol{b}_{1})X_{ij} + \exp(\boldsymbol{A}_{ii^{\prime}}^{\prime}\boldsymbol{b}_{2})Y_{ij} \text{ for}\ j \,=\, 1, \dots, m_{i}^{*}\!. $$

The authors use *O*_*L*_(·,·), a symmetric, continuous function on {(*t*,*s*):0≤*t*≤*L*,0≤*s*≤*L*} such that *O*_*L*_(*s*,*t*) is monotonic in *t* if *s* is given and vice versa to derive the following U-statistic-based estimating equations:

$${{\begin{aligned} D_{1}^{*}(\boldsymbol{b}_{1}) = n^{-2} \sum\limits_{i=1}^{n} \left[ \sum\limits_{i^{\prime}=1}^{n} \boldsymbol{A}_{ii^{\prime}} \frac{1}{m_{i}^{*}} \sum\limits_{j=1}^{m_{i}^{*}} \frac{\Delta_{ij}^{X} O_{L_{1}} \{ X_{ij}, X_{ii^{\prime}j}(\boldsymbol{b}_{1})\}}{\hat{G}_{1}\{X_{ij} \wedge L_{1} \}} \right], \end{aligned}}} $$

$${{\begin{aligned} D_{2}^{*}(\boldsymbol{b}) = n^{-2} \sum\limits_{i=1}^{n} \left[ \sum\limits_{i^{\prime}=1}^{n} \boldsymbol{A}_{ii^{\prime}} \frac{1}{m_{i}^{*}} \sum\limits_{j=1}^{m_{i}^{*}} \frac{\Delta_{ij}^{Y} O_{L_{2}} \{ Z_{ij}, Z_{ii^{\prime}j}(\boldsymbol{b})\}}{\hat{G}_{2}\{Z_{ij} \wedge L_{2}\}} \right], \end{aligned}}} $$

where $\hat {G}_{1}$ and $\hat {G}_{2}$ are Kaplan-Meier estimators of the survival function of the censoring time *G*(·) based on the data $\{(X_{i1}, 1-\Delta _{i1}^{X}), i=1, \dots, n\}$ and $\{(Z_{i1}, 1-\Delta _{i1}^{Y}), i=1, \dots, n\}$, respectively, and *L*_1_<*τ*_*c*_ and *L*_2_<*τ*_*c*_ are limits that ensure respect for the support of *G*(·). The bivariate functions in the estimating functions, $O_{L_{1}}$ and $O_{L_{2}}$ are not necessarily the same. Current implementations used *L*_1_=*L*_2_=*L* and *O*_*L*_(*s*,*t*)= log[ min{max(*t*,*s*),*L*}]− log(*L*). The regression coefficient estimators, $\hat {\boldsymbol {\beta }}_{1}$ and $\hat {\boldsymbol {\beta }}_{2}$ can be obtained by inductively solving $D_{1}^{*}(\boldsymbol {b}_{1}) = 0$ and $D_{2}^{*}((\hat {\boldsymbol {\beta }}_{1}', \boldsymbol {b}_{2}^{\prime })^{\prime })=0$. Note that the smooth and monotonic nature of the estimating equations guarantees a unique solution, a property not possessed by Chang’s method. Moreover, Lee et al. proved the weak convergence of $n^{1/2}(\hat {\boldsymbol {\beta }} - \boldsymbol {\beta })$ to a mean zero normal distribution with variance that can be consistently estimated by $\hat {\Sigma }_{\hat {\boldsymbol {\beta }}}^{-1} \hat {\Omega }^{*} (\hat {\Sigma }_{\hat {\boldsymbol {\beta }}}^{-1})'$ where the definitions of $\hat {\Sigma }_{\hat {\boldsymbol {\beta }}}$ and $\hat {\Omega }^{*}$ can be found in [3].

## Results

We use a subset from the South Verona Psychiatric Case Register (PCR) [8] to illustrate functions in BivRec for data exploration, visualization and analysis in R. Our PCR sample contains data on patients’ care and break periods and disease-related and socioeconomic factors such as age at disease onset, education level, and sex from 336 patients with schizophrenia or related disorders with their conditions first recorded between 1981 and 1995 in South Verona, Italy. We focus on two covariates, one categorical, EDU, and one continuous, Age10, that were previously studied in [3]. These correspond to the education level and age at onset (in 10 years), respectively. We also show a simulated data set and how to simulate data with a function in BivRec in a later section.

### Data preparation

Even though bivariate alternating recurrent data may be displayed in a wide or long format, in line with various longitudinal methods, the BivRec package requires that the data is in a long format, with possibly multiple rows for each participant, reflecting the number of episodes that the participant experienced. In addition to a set of baseline covariates (which repeats for each row if a participant has more than one row in the data), the long format data should have six columns corresponding to $i,j,X_{ij},Y_{ij},\Delta _{ij}^{X}$, and $\Delta _{ij}^{Y}$ which are defined in the previous section and can be specified in the statements of a data object function bivrecSurv() by “id=”, “episode=”, “xij=”, “yij=”, “d1=”, and “d2=”, respectively. See Table 1 for the detailed definitions of these arguments. All the functions for data exploration and analysis in BivRec use the data object created with this function. We used the PCR data to create a bivrecSurv() object as follows (see the package manual [9] for further details):

**Table 1:** Arguments and compatible standard functions for function bivrecSurv()

Argument	Description
id	Vector of subject’s unique identifier.
episode	Vector indicating the pair or episode number (j) for a subject (i); this will determine order of events for each subject.
xij	Vector with the lengths of time spent in event of Type I for individual i in episode j.
yij	Vector with the lengths of time spent in event of Type II for individual i in episode j.
d1	Vector of censoring indicator corresponding to Type I gap times (xij); d1 = 1 for uncensored, and = 0 for censored gap times.
d2	Vector of censoring indicator corresponding to Type II gap times (yij); d2 = 1 for uncensored, and = 0 for censored gap times. Note that in the last episode, yij is always censored (i.e., d2 = 0).
Compatible functions:	plot()


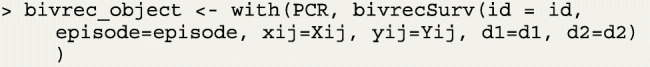


Note that in practice, the input data may have quality issues such as unequal length of variables, negative values for the gap time variables (xij and yij), gaps or non-integers in the episode variable within a subject (e.g., *j*=1,3,4 or *j*= a, b, c), unreasonable values in the censoring indicators (e.g., (d1, d2) = (0, 1) for any episode or (d1, d2) = (1, 1) for the last episode), or no subjects having any uncensored episodes observed (i.e., all d2 = 0). In these cases, the user will get an error message such as “Error: Data not cleaned” with possibly more details to help pinpoint the problem. However, missing values are allowed in the input data even though only subjects with complete data will be used in any subsequent analysis. It is worthwhile to mention that in applications, the time variables may not be observed continuously (e.g., in days); for example, a subject’s censoring event could occur on the same day as the last observed event, causing the situation of (d1, d2) = (1, 1) for the last episode of events (*j*=*m*_*i*_). For these subjects, we suggest users to add a small quantity to the censoring time during the data cleaning process.

### Data exploration

**Fig. 2:**
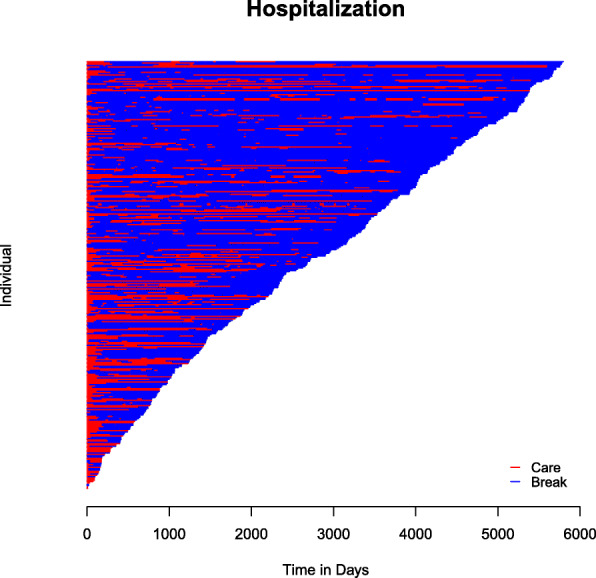
Care and break periods in the South-Verona Psychiatric Case Register (PCR) data sorted by the overall follow-up time of each individual


We begin the exploration of this data set by obtaining a visualization of the care and break periods (Fig. 2) using the plot() function on a bivrecSurv object in the following way:

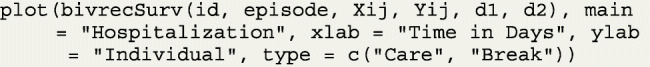


The resulting plot shows that during the study, the majority of the observation time was spent out of the hospital or care facility, as accentuated by the larger portions of blue in the graph.

**Fig. 3:**
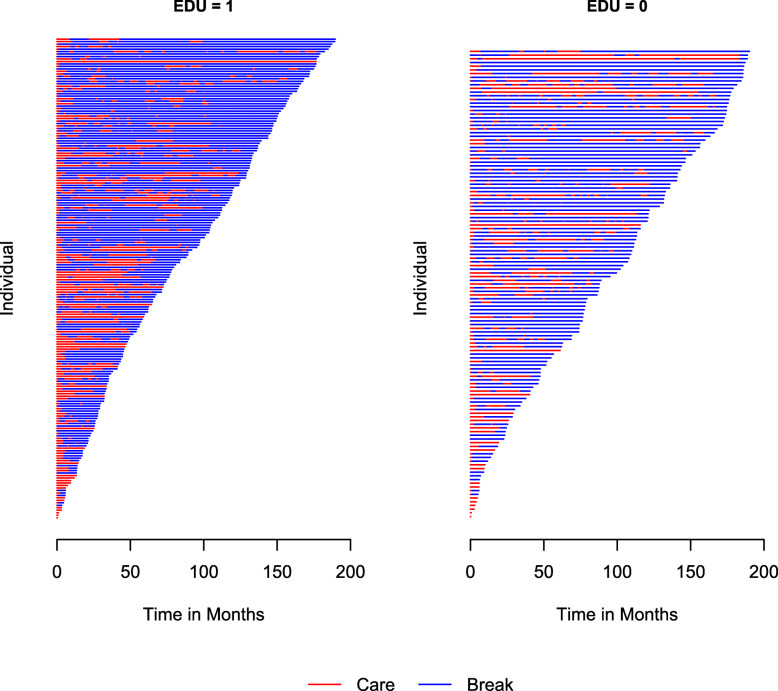
Care and break periods in the PCR data stratified by education (1 = secondary education or higher; 0 = less than secondary education)


The data can also be viewed in subgroups defined by a categorical covariate of six or less levels if a user specifies such a covariate using the by argument of plot(). An example of this feature is shown by looking at times in and out of care based on education levels in the PCR data set. Figure 3 shows the resulting subgroup plots for the levels of the variable EDU, an indicator with values of one for participants with secondary or higher education levels and zero otherwise. Note that in this example, the covariate Age10 is automatically dropped from the by statement, since the function detects that Age10 is possibly a continuous variable. In addition, 10 subjects had missing values for the EDU variable, which is reflected in a message letting the user know only 326 of the subjects were used instead of the full 336 sample.

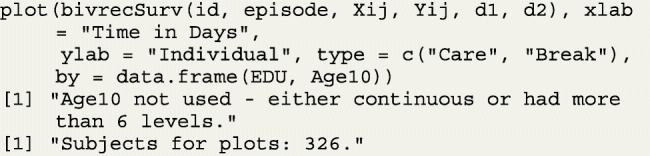


The “bluer” shade of the left panel compared to the one on the right in Fig. 3 indicates that patients with higher education level might have longer break periods than those with less education. Thus, considering education as a factor that affects the re-hospitalization process is reasonable. Note that more than one categorical covariate can be specified in the by argument, but subgroup plots are created for each covariate separately. If subgroups defined by the combinations of multiple categorical variables (e.g., sex and race) are desired, users can define a new categorical variable (with ≤6 levels) before applying the plot() function.

### Nonparametric analysis

We use the function bivrecNP() to estimate the joint distribution $F_{X^{0}, Y^{0}}(x, y)$ for all combinations of values given by the options u1 and u2. The non-negative weight in the nonparametric estimator in Equation (1), *a*_*i*_=*a*(*C*_*i*_), is specified by the option ai. For the data example, we use a simple unit weight *a*(*C*_*i*_)=1 by setting ai=1 (the default). If one sets ai=2, the weight will be the censoring time of each subject, *a*(*C*_*i*_)=*C*_*i*_. The function will automatically estimate the marginal survival probability for Type I gap times $S_{X^{0}}$ for all distinct and uncensored recurrence times. We set conditional=TRUE to request the conditional distribution estimate for Type II gap times given that Type I gap times fall into a certain interval specified by the given.interval argument. In the following example, we set given.interval=c(100, 500) to estimate $F_{Y^{0}|X^{0}}(y|100 \leq X^{0} \leq 500)$, and the confidence level of the point-wise confidence intervals is set by level=0.99 as an illustration that the confidence level can be changed to values different than 0.95. Table 2 and the package manual [9] provide further details of the function bivrecNP().

**Table 2:** Arguments and compatible standard functions for function bivrecNP()

Argument	Description
response	A response object of the bivrecSurv class.
level	The confidence level for the point-wise confidence interval; must be between 0.50 and 0.99; the default value is 0.95.
ai	Value 1 or 2 to indicate which weight function to use in the nonparametric estimator; 1 indicates that the weights are 1 for all subjects, *a*(*C*_*i*_)=1 (default); 2 indicates that the weight is the subject’s censoring time, *a*(*C*_*i*_)=*C*_*i*_.
u1	A vector (or single number) of time values to be used for the estimation of the joint cdf, Pr(*X*^0^≤u1,*Y*^0^≤u2).
u2	A vector (or single number) of time values to be used for the estimation of the joint cdf, Pr(*X*^0^≤u1,*Y*^0^≤u2).
conditional	A logical value. If TRUE, this function will calculate the conditional cdf for the Type II gap time given an interval of the Type I gap time and the bootstrap standard error and confidence interval at the specified confidence level; the default is FALSE.
given.interval	A vector c(v1, v2) that must be specified if conditional=TRUE. The vector indicates an interval for the Type I gap time to be used for the estimation of the cdf of the Type II gap time given this interval.
	If given.interval=c(v1, v2), the function calculates Pr(*Y*^0^≤*y*|v1≤*X*^0^≤v2). The given values v1 and v2 must be in the range of gap times in the estimated marginal survival.
Compatible functions:	plot(), head(), print()


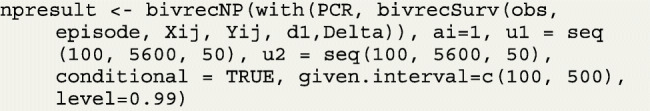


Using the function head() we show a snapshot of all the output elements. To see the entire data frame for all the output elements, use the R function print(). The individual output elements (joint_cdf, marginal_survival, and conditional_cdf) can also be retrieved using the $ operator following the output object.


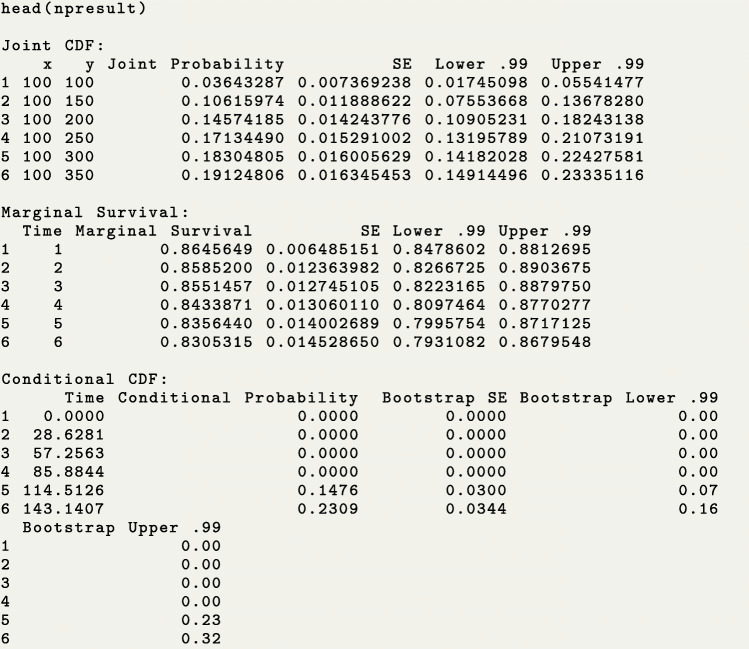


We use the plot() function on the resulting object to simultaneously generate plots for all the estimated distributions. To individually plot the joint *cdf*, the marginal survival probability and the conditional *cdf*, one can use the plotJoint, plotMarg and plotCond functions, respectively.


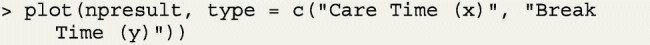


**Fig. 4:**
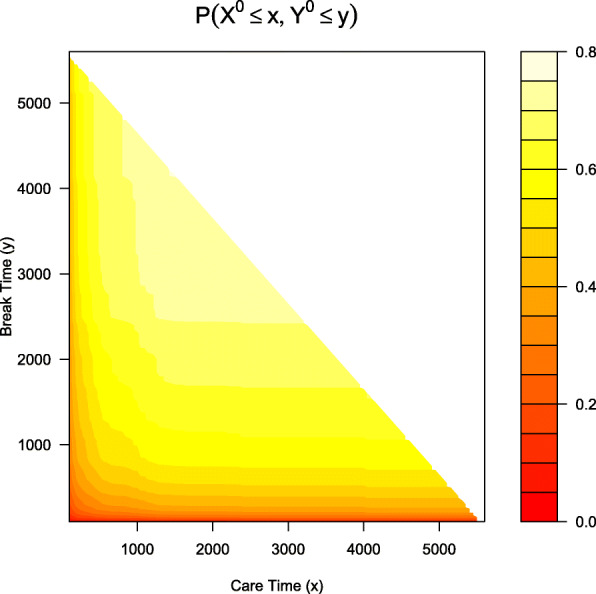
Contour plot of the joint *cdf* of the care and break periods for combinations that meet the condition *x*+*y*≤*τ*_*c*_, where *τ*_*c*_=5697

Figure 4 is the contour plot of the joint *cdf* in the half-plane where estimations meet the condition *x*+*y*≤*τ*_*c*_, for *τ*_*c*_=5697. The joint *cdf* shows that, for instance, the probability that participants were in care for 2000 days or less and out of care for 3000 days or less is over 0.70. Note that the choices of u1 and u2 in the code will determine if the plot of the joint distribution is drawn on a half plane or not. For instance, if we had coded u1=seq(100, 2000, 50) and u2=seq(100, 3000, 50), then all the (*x*,*y*) pairs defined by u1 and u2 would be estimable because max(u1)+ max(u2)≤*τ*_*c*_. In addition, the resulting object (npresult in this example) will show values of NA for the joint probability for any combination of u1 and u2 that does not meet the *x*+*y*≤*τ*_*c*_ condition.

**Fig. 5:**
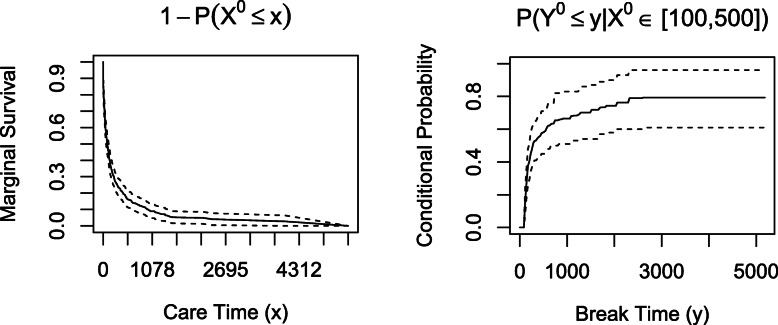
Plots of the marginal survival probability of the care period and the conditional *cdf* of the break period given the care period

The line plots of the estimated marginal survival probability and conditional *cdf* (with corresponding 99% confidence intervals in this example) are shown in Fig. 5. The left panel shows that care times shorter than a year and a half are the most likely as the survival probability drops sharply before 500 days. With this in mind, we look at the conditional *cdf* plot for patients who received between 100 and 500 days of care and conclude that the probability that those patients spent 3000 or fewer days out of care was close to 80%. The bivrecNP() function with conditional=TRUE specification for the PCR data produced results in 248.39 seconds (4.14 minutes, using an AMD Ryzen 5 3500U with Radeon Vega Mobile Gfx 2.10 GHz processor with 16 GB RAM for this and other analyses in this paper). Note that the computing time would be shorter if a less fine grid was specified for u1 and u2. The visualizations for the results, shown in Figs. 4 and 5 ran in less than 2 seconds each.

### Semiparametric regression

The function bivrecReg() provides point estimates, standard error estimates, an estimated variance-covariance matrix and confidence intervals at a specified confidence level for the effects of covariate(s) on the two types of gap times, based on the Chang’s method and Lee et al.’s method. Using the PCR data, we show how to estimate the effects of the education and age of disease onset on the two types of gap times $X_{ij}^{0}$ (care time) and $Y_{ij}^{0}$ (break time) by fitting the AFT model using the function bivrecReg(). We again need to specify a bivrecSurv object as the response, but now we add the covariates to the right hand side of the formula (for further details on the syntax of this function see Table 3 and the reference manual of this package [9]). Note that only baseline (i.e., time-invariant) covariates can be fit in the AFT model in Eq. (2), otherwise, user will get the message, ~Error: Time-varying covariates not allowed.~ We use the function’s default estimation method, Lee et al.’s method, to obtain estimates and Wald confidence intervals based on the asymptotic standard error estimate. This method can also be explicitly specified by the option method=~Lee.et.al~. The messages shown while the function is running and the model fitting output from summary() are as follows.

**Table 3:** Arguments and compatible standard functions for function bivrecReg()

Argument	Description
formula	A formula with a bivrecSurv object on the left of a ’ ∼’ operator as response, and the covariate(s) on the right.
data	A data frame that includes all the variables listed in the formula.
method	A string indicating which method (“Lee.et.al” or “Chang”) to estimate the effects of covariates; the default is “Lee.et.al”.
Compatible functions:	summary(), vcov(), coef(), confint(), print()


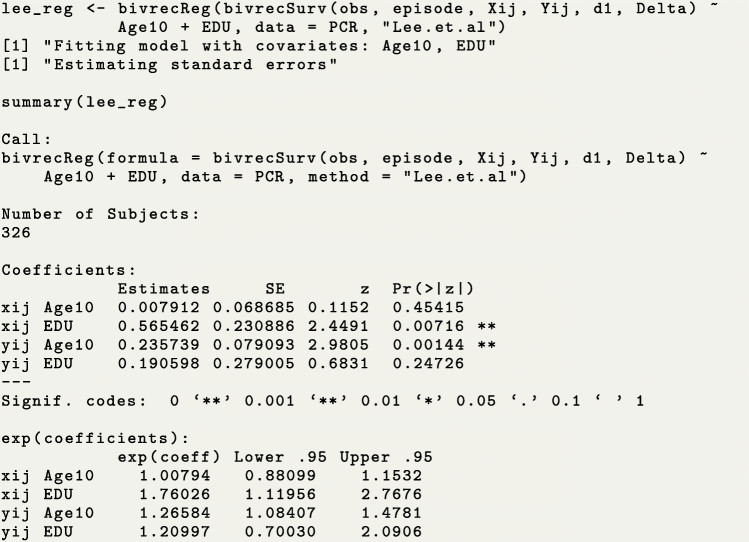


We show the confidence interval for education and the full variance-covariance matrix using the functions confint() and vcov(), respectively. Similar to the standard R function confint(), one can specify the confidence level and an individual parameter of interest. If needed, one could also see coefficient results using the function coef().


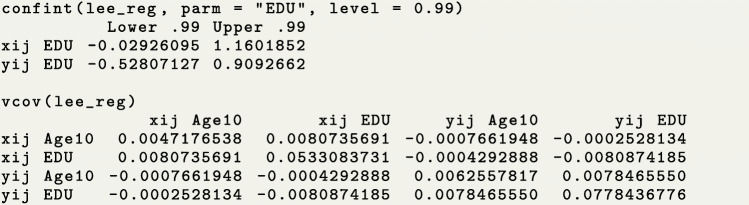


Based on the above results, we conclude that patients in the secondary education or higher (EDU=1) group tended to have 1.76 (=*e*^0.56^, p-value =0.007) times longer care periods and 1.21 (=*e*^0.19^, p-value >0.05) times longer break periods than patients with less education (EDU=0) after adjusting for covariate Age10. In addition, a ten year delay in age of disease onset was associated with a 1.27-fold (=*e*^0.24^, p-value =0.001) increase in the length of break period and a minimal change in the care period of less than 1% (=*e*^0.007^, p-value >0.05), holding EDU constant.

We also fit the AFT model using Chang’s method for point estimation along with Parzen’s resampling algorithm to obtain standard error estimates and construct Wald confidence intervals. The results are as follows.


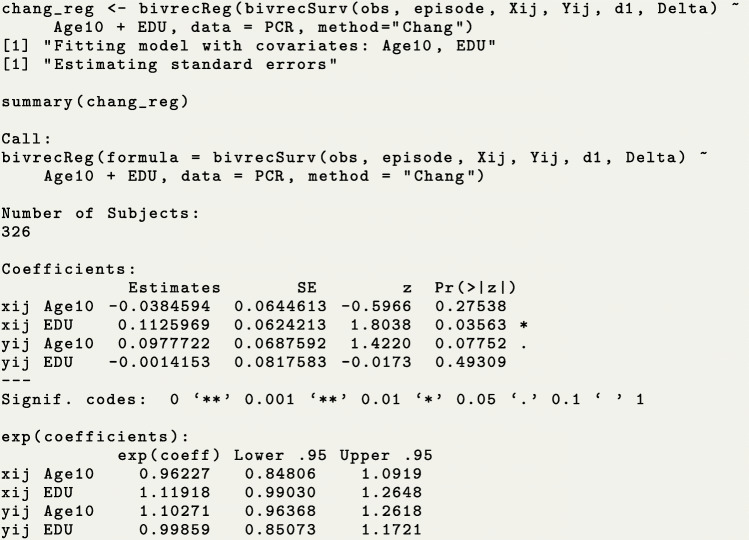


It is important to point out that due to the resampling algorithm needed to obtain variances, applying Chang’s method to the PCR data (*n*=326 after omitting missing values for EDU) led to substantially longer computing times than applying Lee et al.’s method (56 minutes vs. 67 seconds). Similarly, applying these methods to a simulated data set with a smaller sample size, *n*=150, led to a much shorter computing time (602 and 16 seconds for the two methods, respectively). In addition, as discussed earlier, the convergence of Chang’s method is not guaranteed for any dataset, and when the convergence is not achieved, users get the following error message, ~Error: Max Iterations reached. Did not converge.~ For these reasons, we recommend to use the default ~Lee.et.al~ method for fitting the AFT model. For a comparison of the estimation bias, standard error, and coverage probability between these two methods with more extensive simulation studies, see [3]. Nonetheless, we show how to use function simBivRec() provided in the BivRec package to simulate a bivariate alternating recurrent event data. This provides a way for users to perform their own simulation studies.

Following [3], in the example below, we show how to simulate a dataset with a categorical covariate a1 from a binomial distribution with success probability 0.5 and a continuous covariate a2 from a uniform (0,1) distribution using the function simBivRec(). This function has options to simulate data based on the various scenarios outlined in [3]. As an example, we set the parameters in the same way as for the scenario presented in the top panel of Table 3 in [3]: the sample size is *n*=150 (nsize=150); the regression coefficients for the effect of covariate a1 on the Type I and Type II gap times are set as beta1=c(0.5, 0.5); those for covariate a2 are set as beta2=c(0, -0.5); and the support of the uniform distribution (0,*τ*_*c*_) for the censoring time is set with tau_c=63, which yields censoring rate of 15% for the first bivariate gap time pairs, on average. Additional parameters for the within-subject correlation structure of the gap times are set through the option, set=1.1, meaning the first parameter setting of simulation scenario 1 in [3].


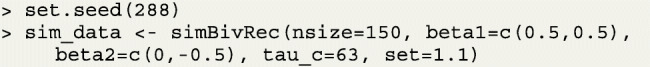


The first few lines of the simulated data are shown as follows, where id is the subject ID (which can take both numerical and string values in the package), epi is the episode number of a gap time pair, xij and yij are the *j*^th^ episode of the observed Type I and Type II gap times of subject *i*, respectively, ci is the overall censoring time of subject *i*, d1 and d2 are their corresponding censoring indicators, and a1 and a2 are two baseline covariates.


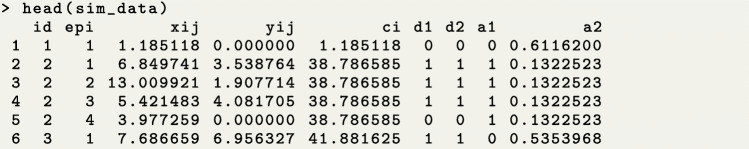


## Conclusions

Despite the growing need and interest in the study of recurrent event data along with the development of R packages for its analysis such as survrec [10], reda [11] and reReg [12], software for analyzing bivariate alternating recurrent events has been lacking. In this paper, we reviewed nonparametric and semiparametric regression methods for gap times between alternating recurrent events and demonstrated how to use the BivRec package that we developed in R to perform these analyses. We also demonstrated BivRec’s capabilities for data visualization and simulation.

There is still a need for further package development such as additions of univariate recurrent gap times (i.e., gap times of recurrent events of the same type) methods as the degenerated case of the bivariate alternating gap times data such as the seminal nonparametric work by Wang and Chang (1999) [5] and the subsequent semiparametric regression methods by various authors [2, 13–16]. Finally, some competing, intensity-based models for bivariate alternating recurrent event data, which are not included in our package, can be found in [17] and [18].

## Availability and requirements

**Project name:** Bivariate Alternating Recurrent Event Data Analysis

**Project home page:** https://cran.r-project.org/web/packages/BivRec/index.html

**Operating system(s):** Platform independent

**Programming language:** R

**Other requirements:** R 3.5.0 or higher

**License:** GPL-3

**Any restrictions to use by non-academics:** None

## Data Availability

Requests for the South Verona PCR data can be made by contacting the principal investigator of the WHO Collaborating Centre for Research and Training in Mental Health and Service Evaluation at https://www.dnbm.univr.it/?ent=bibliocr&id=254&tipobc=6&lang=en. R source code is available at https://github.com/SandraCastroPearson/BivRec.

## Abbreviations

PCR: Psychiatric Case Register
AFT: Accelerated Failure Time
